# Statistical Design of Sustained-Release Tablet *Garcinia cambogia* Extract and Bioconverted Mulberry Leaf Extract for Anti-Obesity

**DOI:** 10.3390/pharmaceutics12100932

**Published:** 2020-09-29

**Authors:** Hye-Jin Lee, Young-Guk Na, Mingu Han, Thi Mai Anh Pham, Hyeonmin Lee, Hong-Ki Lee, Chang-Seon Myung, Joo-Hui Han, Jong-Seong Kang, Kyung-Tae Kim, Cheong-Weon Cho

**Affiliations:** 1 College of Pharmacy and Institute of Drug Research and Development, Chungnam National University, 99 Daehak-ro, Yuseong-gu, Daejeon 34134, Korea; haejin101@naver.com (H.-J.L.); youngguk@cnu.ac.kr (Y.-G.N.); linuxfalcon@naver.com (M.H.); phammaianhdkh68@gmail.com (T.M.A.P.); gusals2218@naver.com (H.L.); hk_lee@cnu.ac.kr (H.-K.L.); cm8r@cnu.ac.kr (C.-S.M.); han5621@cnu.ac.kr (J.-H.H.); kangjss@cnu.ac.kr (J.-S.K.); 2 Department of Food and Nutrition, Dong-Eui University, 176 Eomgwangno, Busanjin-gu, Busan 47340, Korea; kimkkt@deu.ac.kr

**Keywords:** *Garcinia cambogia*, mulberry leaf, sustained-release tablet, response surface design

## Abstract

Obesity is a major health concern worldwide, and it is leading to worsening disease morbidity and mortality. Herbal supplements and diet-based therapies have attracted interest in the treatment of obesity. It is known that *Garcinia cambogia* (GA) and mulberry leaf, which contain polyphenols, have anti-obesity activity. Herein, we developed a combined tablet consisting of GA extract and bioconverted mulberry leaf extract (BMUL) using a statistical design approach. The ratio and amount of sustained polymers were set as factors. In the cell study, the combination of GA and BMUL showed synergistic anti-obesity activity. In a statistical model, the optimized amounts of hydroxypropyl methylcellulose 2208 (HPMC 2208) and polyethylene oxide 303 (POLYOX 303) were 41.02% and 58.98%, respectively. Additionally, the selected ratio of microcrystalline cellulose (MCC) was 0.33. When the release, hardness, and friability of the GABMUL tablet were evaluated, the error percentages of the response were lower than 10%. This indicates that the GABMUL tablet was successfully prepared.

## 1. Introduction

Obesity is a major health concern worldwide, and it is leading to worsening disease morbidity and mortality [1]. For the treatment of obesity, there are six major drug classes: orlistat, phentermine, phentermine/topiramate extended release, lorcaserin, naltrexone/bupropion sustained release, and liraglutide [2]. However, some patients may experience side effects, like constipation, dizziness, headache, and abdominal pain [3]. Thus, herbal supplements and diet-based therapies have attracted interest in the treatment of obesity [4]. In particular, it has been reported that polyphenol micronutrients modulate physiological and molecular pathways in energy metabolism, adiposity, and obesity [5]. It has been reported that a large amount of polyphenols is contained in cocoa, coffee, green tea, mulberry, and garcinia [6,7,8]. Therefore, a combined strategy of herbal medicine will be an effective strategy for the treatment of obesity. Thus, here, we developed a combined tablet consisting of *Garcinia cambogia* extract (GA) and mulberry leaf extract (MUL). To improve the bioactivity of herbal products, bioconversion techniques have been widely used [9,10]. Bioconversion, such as microbial fermentation and enzyme treatment, has led to the improvement of bioactivity by enhancing the production of active ingredients. According to Jung et al., bioconverted MUL (BMUL) has improved the ability to decrease fasting blood glucose levels, tolerate glucose, decrease blood HbA1C levels, and increase plasma insulin compared with MUL [11]. Additionally, it has been reported that BMUL has shown antidiabetic effects in diabetic rats [12]. Thus, here, we used BMUL rather than MUL for further experiment.

A sustained-release (SR) tablet could control the drug release; thus it maintains drug efficacy over a long period. The blood concentration of an SR drug would be maintained at therapeutic concentration throughout the dosing interval. In addition, the SR drug delivery system has many advantages, including improved patient compliance, reduced toxicity due to overdose, and minimized drug accumulation with chronic dosing. However, it is difficult to develop an SR tablet of an herbal medicine that can be administrated once a day because of difficulty in controlling the release and analyzing the indicator component of the herbal medicine. Thus, an SR tablet of GA and BMUL has not yet been developed.

Statistical design approaches are a useful tool to optimize a tablet, and it has been widely applied for process optimization. In particular, a response surface design (design of experiments (DoE)), an advanced statistical design approach, has been widely used for process optimization, and it reveals the relationship between several variables and response variables. This model has been widely applied to understand the interaction between factors (composition of tablets and type of excipient) and the quality of the tablet.

Herein, we aimed to develop an SR tablet containing GA and BMUL that exhibits a constant drug release. A response surface design was used to study the effect of the ratio and amount of sustained polymers on responses, such as percentage of drug released over time.

## 2. Materials and Methods

### 2.1. Materials

GA and BMUL were obtained from MSC (Yangsan, Korea). In this study, fermented MUL (BMUL) was used according to the literature previously reported [13]. In brief, *Morus alba* L. was extracted in water at 121 °C for 3 h. One percent (*w*/*v*) enzyme (Pectinex^®^, Novozymes Korea, Seoul, Korea) was added, and they were fermented at pH 5.0 at 45 °C for 15 h. Then, they were lyophilized using a freeze dryer. Hydroxypropyl methylcellulose 2208 (HPMC, 15,000 mPa⋅s) was purchased from Shin-Etsu ChemCo., Ltd. (Tokyo, Japan). Polyethylene oxide 303 (POLYOX, 8700 mPa⋅s) was provided by Colorcon, Inc. (Harleysville, PA, USA). Avicel PH102 (microcrystalline cellulose) was purchased from FMS Corporation (Philadelphia, PA, USA). Aerosil 200 (silicon dioxide) was provided by Evonik Industries AG (Essen, Germany). PVP K-30 (polyvinylpyrrolidone) was provided by Ashland Inc. (Covington, KY, USA). Magnesium stearate was purchased from FACI (Genova, Italy). High-performance liquid chromatography (HPLC)-grade acetonitrile (ACN) and methanol (MeOH) were obtained from J T baker (Phillipsburg, NJ, USA). Dulbecco’s modified Eagle’s medium (DMEM), fetal bovine serum (FBS), bovine calf serum, phosphate-buffered saline (PBS), trypsin-EDTA, and penicillin/streptomycin were obtained from Gibco BRL (Grand Island, NE, USA). Dexamethasone, 3-isobutyl-1-methylxanthine (IBMX), insulin, Oil Red O, isopropanol, epigallocatechin gallate (EGCG), digitonin, and 3-(4,5-dimethylthiazol-2-yl)-2,5-diphenyltetrazolium bromide (MTT) were from Sigma-Aldrich (St. Louis, MO, USA).

### 2.2. Total Polyphenol Content (TPC) Assay

For the determination of polyphenol, an indicator substance of BMUL, a slightly modified total polyphenol content assay was conducted according to Korean Herbal Pharmacopoeia (Folin–Ciocalteu assay) [13]. In brief, 0.5 mL of the sample was located in the tube, and 0.1 mL of the Folin–Ciocalteu reagent was added in the tube. After the vortex, 5 mL of 15% sodium carbonate was added in the sample in dark condition. After 2 min of incubation at room temperature, the absorbance of the sample was observed at 715 nm using a Ultraviolet–visible (UV–VIS) spectrophotometer (S-3100, Sinco, Seoul, Korea). Pyrogallol (Toronto Research Chemicals, Toronto, ON, Canada) was used to determine the standard curve, and the total polyphenol content (TPC) was expressed in pyrogallol equivalents.

### 2.3. HPLC Analysis

Hydroxycitric acid, an indicator substance of GA, was analyzed by the HPLC system with a UV detector (Nexera-i MT series, Shimadzu Corp., Kyoto, Japan). Hydroxycitric acid was detected using a C18 column (Kinetex C18, 250 × 4.6 mm, 5 μm), which was maintained at 25 °C. The mobile phase was distilled water (10 mM sulfuric acid), and the flow rate was 0.8 mL/min. The wavelength was set at 210 nm, and the injection volume was 20 µL.

### 2.4. Anti-Obesity Activity of GA and BMUL

Before the preparation of a GABMUL tablet, the synergistic interaction between GA and BMUL was evaluated using 3T3-L1 cells.

#### 2.4.1. Cell Culture and Differentiation

Mouse 3T3-L1 cells were purchased from the American Type Culture Collection (ATCC, Rockville, MD, USA). The 3T3-L1 cells were cultured in DMEM with 10% bovine calf serum at 37 °C in a 5% CO_2_ atmosphere. After 2 days (designated day 0), differentiation was induced by changing the medium with 10% FBS, 0.5 mM IBMX, 1 μM dexamethasone, and 1 μg/mL insulin for 3 days. The medium was replaced with DMEM containing 10% FBS and 10 μg/mL insulin for the following 2 days, and the cells were then maintained in DMEM with 10% FBS for 2 days. The 3T3-L1 cells were treated with or without compounds from day 0 and maintained in the medium throughout the experiment.

#### 2.4.2. Oil Red O Assay

After differentiation, the cells were stained with Oil Red O to detect lipid accumulation in adipocytes. The cells were washed with PBS, fixed with 4% paraformaldehyde for 1 h, and then stained with Oil Red O in 60% isopropanol for 20 min. After lipid droplets were stained, the Oil Red O staining solution was removed, and the plates were rinsed with water. Images of the stained lipid droplets were collected on a microscope (Olympus IX71, Tokyo, Japan). Finally, the dye retained in the cells was eluted with isopropanol for 10 min and quantified by measuring the absorbance at 520 nm using a microplate reader (Tecan Group Ltd., Männedorf, Switzerland).

#### 2.4.3. Cell Viability Assay

For the cytotoxicity test of compounds in 3T3-L1 preadipocytes, MTT assay was performed. When the 3T3-L1 preadipocytes reached 70% confluence, the cells were incubated with a serum-free medium for another 24 h, and then treated with various compounds for the indicated concentration and 100 μg/mL digitonin as a cytotoxic control. After 24, 48, and 72 h, the MTT reagent (5 mg/mL) was added to each well for 3 h at 37 °C in a 5% CO_2_ atmosphere. The cells were treated with 200 μL DMSO, and the absorbance at 565 nm was measured using a microplate reader (Tecan Group Ltd., Männedorf, Switzerland). For the cell proliferation test of compounds in 3T3-L1 adipocytes, CCK-8 assay was performed. After termination of the compounds in the 3T3-L1 adipocytes, the CCK-8 reagent was treated in each well for 1.5 h. After incubation, the absorbance was measured at 450 nm using a microplate reader.

### 2.5. Preparation of the GABMUL Tablet

The tablets were prepared with different sustained polymers (HPMCs and POLYOXs) using the wet granulation method (Table 1). Microcrystalline cellulose (Avicel^®^ PH102) was used as fillers. For the preparation of the GABMUL tablet, ethanol and magnesium stearate (FACI, Genoa, Italy) were used as solvent and lubricant, respectively. Aerosil^®^ 200 was selected as desiccant. In brief, all the ingredients were mixed for 10 min, and ethanol was added for granulation. They were dried at 40 °C and sieved using a 20-mesh sieve. Then, the lubricants were added and mixed for 3 min. They were compressed using a single punch column tablet press (Korsch EK0, Korsch AG, Berlin, Germany) equipped with a round-shape punch (10 mm) and die set at a fixed compression force. The tablet was coated using an Opadry AMB II for the prevention of dampness (3% of the tablet weight). The tablets were coated using a Labcoat™ M coater (O’Hara Technologies Inc., Richmond Hill, ON, Canada) with a spray gun (0.8 mm). The other coating parameters were set as follows: inlet temperature at 55 °C, bed temperature at 45–55 °C, spray rate of 5–7 g/min, pan speed of 5–11 rpm, atomizing air pressure at 0.7 bar, and pattern air pressure at 1.5 bar. The coated tablet was stored at room temperature for further experiments.

### 2.6. Statistical Experimental Design for the GABMUL Tablet

The statistical experimental design and analysis were carried out using Design-Expert^®^ 11 (Stat-Ease Inc., Minneapolis, MN, USA). Here, we used three numeric factors for the statistical experimental design (Table 2). The level of the amount of the sustained polymers, HPMC 2208 and POLYOX 303 (X_1_ and X_2_, %), was set as 0%–100%, and the ratio of the sustained polymers and MCC (X_3_) was set as 0.3–1.2. The acceptable ranges of Y_1_, Y_2_, Y_3_, Y_4_, Y_5_, Y_6_, Y_7_, and Y_8_ were 20–40, 40–60, 80–100, 20–40, 40–60, 80–100, >10 kp, and <0.2%. A total of 19 experiments were conducted. The suitable model for the response was selected by fitting to a linear, cubic, quadratic, special cubic, or quartic model. Additionally, for the evaluation of model acceptance, statistical parameters, including sequential *p*-values, lack of fit, squared correlation coefficient (R^2^), adjusted R^2^, predicted R^2^, and adequate precision, were assessed. In the terminal phase of the statistical modeling, the overlay plot was used to select the optimal composition of the GABMUL tablet.

### 2.7. Characterization of the GABMUL Tablet

The physicochemical properties of the GAMUL tablet were evaluated for hardness and *friability*. For the evaluation of hardness, a tablet hardness tester (TBH 125, ERWEKA GmbH, Heusenstamm, Germany) was used, and the breaking force of the tablet was measured. The measurement was triplicated, and the average value was calculated.

For the evaluation of tablet *friability*, a *friability* tester (PTF-3DR, Pharma Test Apparatebau AG, Hainburg, Germany) was used. The weights (*W*1) of the randomly preselected 10 tablets were measured, and they were placed in the drum. After the rotation (100 times), the weights (*W*2) of the tablets were measured, and % weight loss was calculated by the following equation:

(1) $$Friability\;=\;\frac{W1-W2}{W1}\times 100$$

A *friability* of less than 0.5% was considered acceptable. Although a *friability* of 1% was generally used according to the EU Pharmacopoeia, we applied our internal standards. Of course, even 1% *friability* is acceptable, but for others to have suitable properties for coating and storage, a standard for *friability* of less than 0.2% was set. We evaluated this using the same criteria in other papers.

For investigating responses, the dissolution of each experiment was performed using a dissolution tester (DST-810; Labfine, Anyang, Korea) according to the United States Pharmacopeia (USP) apparatus II method. An amount of 500 mL of distilled water was used as a dissolution medium at 37 °C with 50 rpm paddle speed. A sample (5 mL) was collected at predetermined time points (2, 6, and 12 h) and analyzed with HPLC.

To explain the drug release profiles of the GABMUL tablet, the Korsmeyer–Peppas model was used. The GA and BMUL release profiles were fitted by the following equation [14]:

*M*_*t*_/*M*_∞_ = *kt* (2)

where the *M_t_/M_∞_* is the ratio of drug release at time (*t*) and *k* is a constant. Based on the equation, the diffusion index (*n*) was calculated. The mechanism of drug release could be changed by the *n* value (*n* = 0.45, Fickian release; 0.45 < *n* < 0.89, non-Fickian (anomalous) release; *n* = 0.89, case II release (zero-order release); *n* > 0.89, super case II release) [15].

## 3. Results and Discussion

### 3.1. Anti-Obesity Activity of GA and BMUL

**Figure 1:**
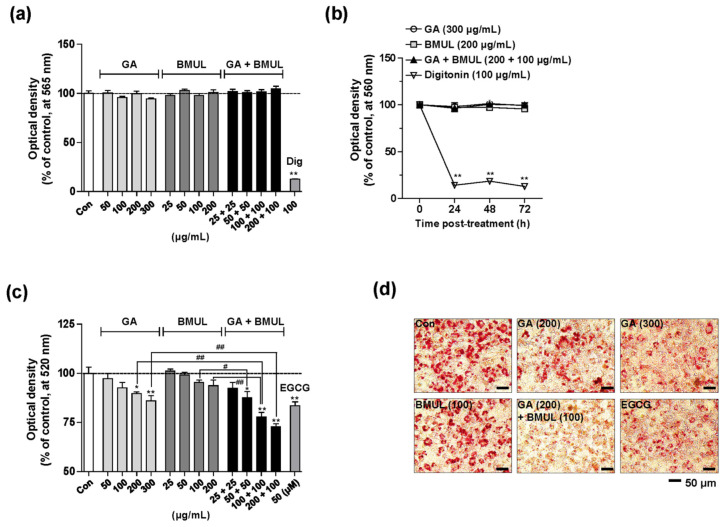
Combined treatment of *Garcinia cambogia* extract (GA) and bioconverted mulberry leaf extract by Pectinex (BMUL) has synergistic effect on inhibition of lipid accumulation in 3T3-L1 cells without cytotoxicity. Cytotoxicity of GA, BMUL, and GA and BMUL mixture (GA + BMUL) in 3T3-L1 preadipocytes. (**a**) Cells were treated with each of the compounds at the indicated concentrations for 24 h, and cell viability was assessed by MTT assay. Digitonin (Dig, 100 μg/mL) was used as positive control for cytotoxicity. (**b**) Cells were treated with the highest concentrations of GA (300 μg/mL), BMUL (200 μg/mL), GA + BMUL (200 + 100 μg/mL), and Dig for 24, 48, and 72 h. (**c**) Effect of mono or combined treatment of GA and BMUL on lipid accumulation in 3T3-L1 adipocytes. Epigallocatechin gallate (EGCG, 50 μM) was used as positive control for adipogenesis. Quantification of lipid accumulation was based on the values of optical density by extracting Oil Red O staining from adipocytes as described in the Methods section. * *p* < 0.05 and ** *p* < 0.01 vs. control; ^#^ *p* < 0.05 and ^##^ *p* < 0.01 vs. each group. (**d**) Representative images of Oil Red O staining. Scale bar: 50 μm.

To determine whether the treatment of various compounds was cytotoxic to 3T3-L1 preadipocytes, MTT assay was performed. GA, BMUL, and a mixture of GA and BMUL (GA + BMUL), except digitonin (Dig, a positive control for cytotoxicity), did not exert any toxic effect in the 3T3-L1 preadipocytes (Figure 1a). The highest concentrations of GA (300 μg/mL), BMUL (200 μg/mL), and GA + BMUL (200 + 100 μg/mL) did not show cytotoxicity until 72 h in the 3T3-L1 preadipocytes (Figure 1b). Since obesity is occurred by differentiation of preadipocytes into mature adipocytes that are loaded with lipid droplets in adipose tissues [16], we examined the inhibitory effect of GA, BMUL, and GA + BMUL mixture on intracellular lipid accumulation in 3T3-L1 adipocytes using Oil Red O assay (Figure 1c,d). Exposure to the various concentrations of GA (50, 100, 200, and 300 μg/mL) decreased lipid accumulation by 2.76%, 7.50%, 10.41%, and 14.09%, indicating that 200 and 300 μg/mL had a significant inhibitory effect. In addition, BMUL (50, 100, and 200 μg/mL) slightly decreased lipid accumulation by 0.87%, 4.64%, and 6.33%, but these were not statistically significant. EGCG was used as a positive control for anti-adipogenic effect [17]. Interestingly, the combination of GA and BMUL (50 + 50, 100 + 100, and 200 + 100 μg/mL) showed greater inhibition of lipid accumulation (12.41%, 22.26%, and 27.22%) compared with GA alone (100, 200, and 300 μg/mL) or BMUL alone (100 and 200 μg/mL), implying a synergistic effect. Thus, the combined tablet of GA and BMUL may show synergistic anti-obesity activity, and a sustained tablet of GABMUL was developed through a statistical design.

### 3.2. Preparation of the GABMUL Tablet

In this study, a response surface design was used for the preparation of the GABMUL tablet. Statistical analysis was conducted by the Design-Expert^®^ 11 software, and the relationships between the model and responses were evaluated. Herein, the % dissolved of drugs at 2 h (Y_1_, Y_4_), 6 h (Y_2_, Y_5_), and 12 h (Y_3_, Y_6_) was measured. For the evaluation of the sustained-release profile of the GABMUL tablet, the % dissolved of drugs was a critical response. In this study, when the amount of polymers increased, the drug was slowly released. This indicates that the sustained polymers are a critical factor in controlling drug release. Additionally, this result has been supported by previous studies [18].

In this study, the responses were fitted using cubic (Y_1_), quadratic (Y_2_, Y_8_), and linear (Y_3_, Y_4_, Y_5_, Y_6_, Y_7_) models (Table 2). Additionally, statistical parameters, such as sequential *p*-value, lack of fit, R^2^, adjusted R^2^, and predicted R^2^, are listed in Table 3. The model was selected by comparing statistical parameters by analysis of variance (ANOVA) (Tables S1–S8). In all the models, the *p*-values were <0.05 and the R^2^ values were >0.9 except Y_4_. However, the precision of Y_4_ was 21.14, which indicates that the model was acceptable in the design space (adequate precision > 4) [19]. This suggests that the fitted models were in acceptable criteria [20,21]. The R^2^ and adjusted R^2^ values correspond to the variability between the model and experimental data [22]. In summary, the models showed good fitting based on the statistical parameters.

**Figure 2:**
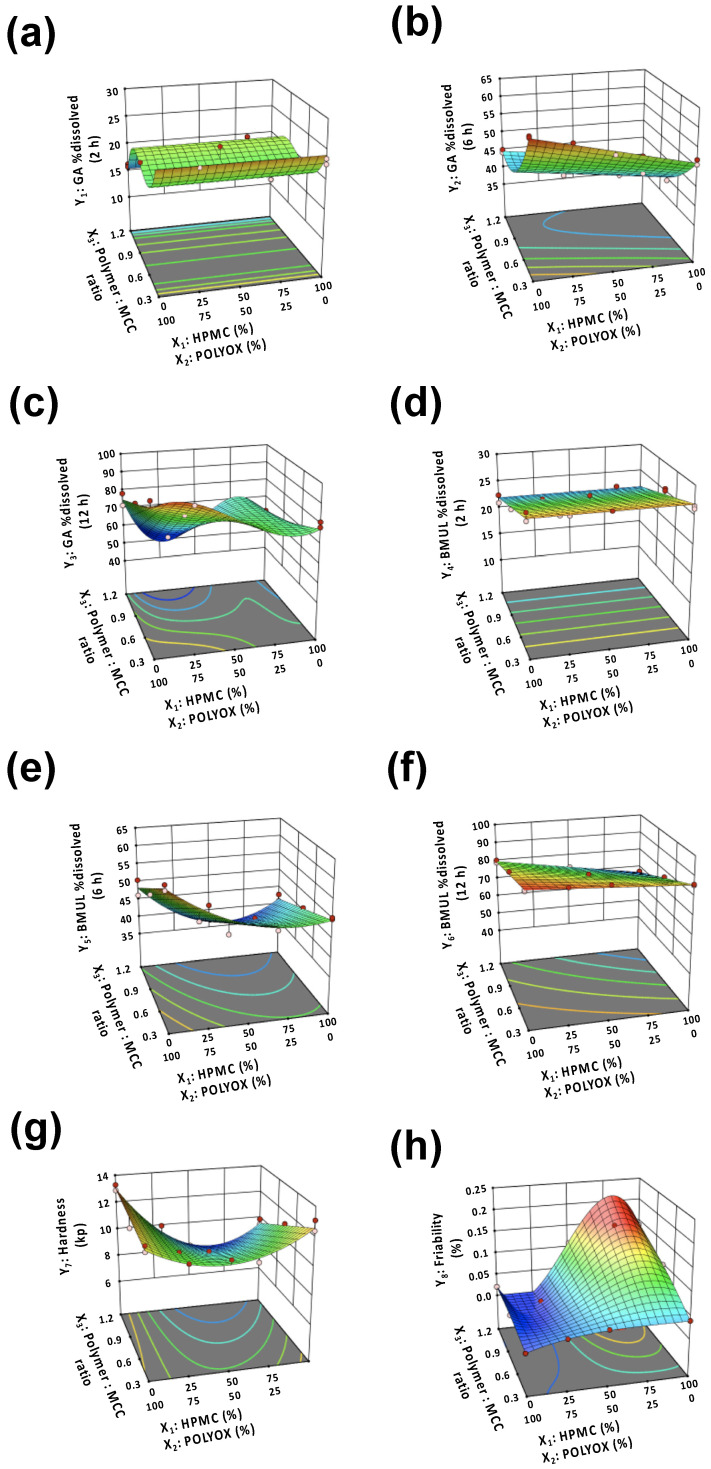
Three-dimensional surface plots of (**a**) Y_1_: % dissolved (2 h) of GA; (**b**)Y_2_: % dissolved (6 h) of GA; (**c**) Y_3_: % dissolved (12 h) of GA; (**d**) Y_4_: % dissolved (2 h) of BMUL; (**e**) Y_5_: % dissolved (6 h) of BMUL; (**f**) Y_6_: % dissolved (12 h) of BMUL; (**g**) Y_7_: hardness; (**h**) Y_8_: friability.

**Figure 3:**
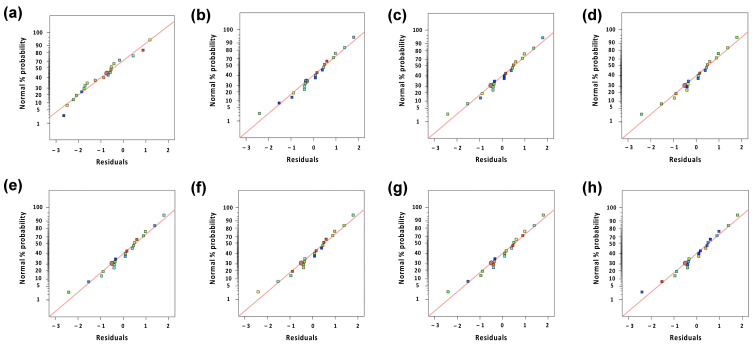
Residual plots. (**a**) Y_1_: GA % dissolved at 2 h; (**b**) Y_2_: GA % dissolved at 6 h; (**c**) Y_3_: GA % dissolved at 12 h; (**d**) Y_4_: BMUL % dissolved at 2 h; (**e**) Y_5_: BMUL % dissolved at 6 h; (**f**) Y_6_: BMUL % dissolved at 12 h; (**g**) Y_7_: hardness; (**h**) Y8: friability.

The interaction plots among the factors and code equations are illustrated and listed in Figure 2 and Table 4. To evaluate the good fitness between the actual values and predicted values, the residuals were assessed (Figure 3). Table 5 shows the aspects and actual values of the responses. In Figure 2a–c, it is shown that the number of polymers did not affect the GA release at 2 h, while the GA release decreased as the amount of polymers increased at 6 and 12 h. Additionally, in similar fashion, the BMUL release decreased as the amount of polymers increased at 6 and 12 h (Figure 2d–f). In general, as the polymer amount increased, the water uptake of the tablet increased, resulting in the proportion of swelling of the tablet. Thus, for the control of the drug release, the amount of polymers would be a critical factor. Additionally, surface erosion of the tablet could be a critical factor for the drug release of the tablet. The use of polymers with high viscosity could retain the erosion of the tablet via the strength of the tablet matrix. In addition, as the amount of HPMC increased, the hardness and friability decreased or increased (Figure 2g,h). This result is similar to that previously reported in the literature of sustained-release tablet with herbal medicine [16].

**Figure 4:**
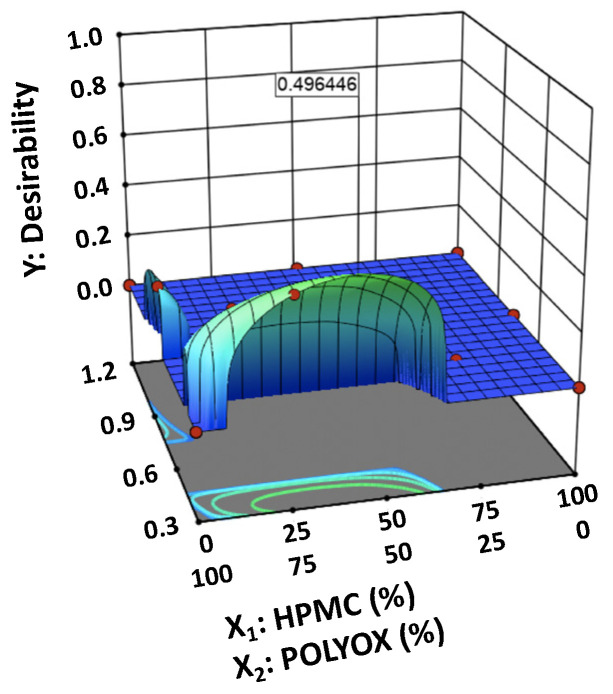
Desirability plot through numerical optimization.

The optimal value of the factors was selected using numerical optimization (Figure 4). The optimized amounts of HPMC 2208 and POLYOX 303 were 41.02% and 58.98%, respectively. Additionally, the selected ratio of polymer:MCC was 0.3.

### 3.3. Characterization of the GABMUL Tablet

Table 6 lists the predicted responses and the actual responses. The predicted values were evaluated close to the actual values. For the evaluation of prediction accuracy, % error was calculated and values of % error were < 10%. This indicates that the GABMUL tablet was successfully optimized and prepared. We consider the GABMUL tablet to be an oral tablet. The half-lives of hydroxycitric acid (an indicator of GA) and polyphenol (an indicator of BMUL) are around 5.4 and 4 h in rats, according to the literature [23,24]. In the release test, the GA + BMUL tablet showed a sustained-release profile. Therefore, it is expected that GABMUL will show sustained release in vivo.

To explain the mechanism of drug release, the Korsmeyer–Peppas model was used, and the parameters are listed in Table 7. The R^2^ values for GA and BMUL were 0.98 and 0.99, respectively. This suggests that the model was acceptable. The *n* values for GA and BMUL were 0.84 and 0.89, respectively. This indicates that GA and BMUL showed the non-Fickian (anomalous) or case II (zero-order) drug release mechanism, respectively. The sustained-release profile of the GABMUL tablet may be due to HPMC and POLYOX.

When the GABMUL tablet contacts with water, HPMC and POLYOX will absorb the water and the surface of the table will swell. In addition, the incorporation of microcrystalline cellulose (water-insoluble polymer) into the hydrophilic matrix could sustain the drug release. Thus, the GABMUL tablet may show a constant release profile in the body system, particularly in the gastrointestinal tract.

## 4. Conclusions

The GABMUL tablet that was developed using a statistical approach released the drug for 12 h. The amount and ratio of sustained polymers were controlled to control the drug release. The optimized composition of GABMUL tablet was consisted of 41.02% HPMC and 58.98% POLYOX as a sustained polymer, and the sustained-release profile of the GABMUL tablet was confirmed. Thus, the statistical approach used in this study would be useful to develop tablets containing the herbal extract. Additionally, this approach could be used to control the drug release of the sustained-release tablet. Moreover, it is expected that the GABMUL tablet will expand the armamentarium of anti-obesity drugs.

## Figures and Tables

**Table 1:** Composition of Garcinia cambogia and bioconverted mulberry leaf extract (GABMUL) tablet.

Premixture		
Components	mg	%
GA	300.0	30.0
BMUL	275.0	27.5
HPMC	0–204.5	0–20.45
POLYOX	0–204.5	0–20.45
Avicel PH102	170.5–288.5	17.05–28.85
Aerosil 200	10.0	1.0
PVP K-30	30.0	3.0
Ethanol	q.s.	q.s.
**Postmixture**		
**Components**	**mg**	**%**
Premixture	990.0	99.0
Magnesium stearate	10.0	1.0
Total	1000.0	100.0

**Table 2:** Factors and responses used in experimental design.

Factors	Levels	
	Low	High
X_1_: HPMC (%)	0	100
X_2_: POLYOX (%)	0	100
X_3_: Polymer:MCC ratio	0.3	1.2
**Responses**	**Acceptable Range (%)**	**Target (%)**
Y_1_: % dissolved of GA (2 h)	20–40	30
Y_2_: % dissolved of GA (6 h)	40–60	50
Y_3_: % dissolved of GA (12 h)	80–100	90
Y_4_: % dissolved of BMUL (2 h)	20–40	30
Y_5_: % dissolved of BMUL (6 h)	40–60	50
Y_6_: % dissolved of BMUL (12 h)	80–100	90
Y_7_: Hardness	>10 kp	-
Y_8_: Friability	<0.5%	-

**Table 3:** Summary of fitted model and statistical analysis.

Responses	Mixture Model	Process Model	*p*-Value	Lack of Fit *p*-Value	R^2^	Adjusted R^2^	Adequate Precision
Y_1_	Mean	Cubic	<0.0001	0.1234	0.9067	0.8880	15.7665
Y_2_	Linear	Quadratic	<0.0001	0.3064	0.9816	0.8745	32.5324
Y_3_	Cubic	Linear	<0.0001	0.5238	0.9511	0.9199	16.2780
Y_4_	Mean	Linear	<0.0001	0.4257	0.8921	0.8858	21.1434
Y_5_	Quadratic	Linear	<0.0001	0.2638	0.9400	0.9169	22.2267
Y_6_	Linear	Linear	<0.0001	0.1907	0.9871	0.9845	56.7055
Y_7_	Quadratic	Linear	<0.0001	0.1604	0.9307	0.9041	18.1049
Y_8_	Cubic	Quadratic	<0.0001	0.8431	0.9991	0.9976	80.6975

**Table 4:** Coded equations of responses according to factors.

Responses	Coded Equations
Y_1_	20.37 + 4.25X_3_ − 0.5743X_3_^2^ − 8.63X_3_^3^
Y_2_	40.44X_1_ + 45.65X_2_ − 6.04X_1_X_3_ − 8.10X_2_X_3_ + 4.69X_1_X_3_^2^ + 7.02X_2_X_3_^2^
Y_3_	67.71X_1_ + 84.54X_2_ − 16.81X_1_X_2_ − 9.84X_1_X_3_ − 9.77X_2_X_3_ − 15.83X_1_X_2_X_3_ + 54.31X_1_X_2_(X_1_ − X_2_) + 107.19X_1_X_2_X_3_(X_1_ − X_2_)
Y_4_	24.42 − 2.47X_3_
Y_5_	45.63X_1_ + 54.74X_2_ − 26.45X_1_X_2_ − 4.13X_1_X_3_ − 6.49X_2_X_3_ − 6.91X_1_X_2_X_3_
Y_6_	76.27X_1_ + 85.01X_2_ − 9.38X_1_X_3_ − 5.36X_2_X_3_
Y_7_	11.08X_1_ + 12.57X_2_ − 9.08X_1_X_2_ − 1.51X_1_X_3_ + 0.4669X_2_X_3_ − 2.47X_1_X_2_X_3_
Y_8_	0.1002X_1_ + 0.0203X_2_ + 0.0467X_1_X_2_ + 0.0502X_1_X_3_ + 0.0052X_2_X_3_ + 0.0373X_1_X_2_X_3_ − 0.0101X_1_X_3_^2^ − 0.0053X_2_X_3_^2^ + 0.3739X_1_X_2_(X_1_ − X_2_) + 0.0445X_1_X_2_X_3_^2^ + 0.4133X_1_X_2_X_3_(X_1_ − X_2_) + 0.0123X_1_X_2_X_3_^2^(X_1_ − X_2_)

**Table 5:** Experimental composition and observed responses through combined design.

Run	Factors			Responses							
	X_1_	X_2_	X_3_	Y_1_	Y_2_	Y_3_	Y_4_	Y_5_	Y_6_	Y_7_	Y_8_
1	0	100	0.3	24.6 ± 2.1	60.9 ± 6.9	97.9 ± 5.8	27.9 ± 3.1	62.1 ± 5.8	90.3 ± 4.1	12.4 ± 1.1	0.01 ± 0.00
2	50	50	1.2	13.8 ± 1.5	41.5 ± 1.1	59.4 ± 6.5	21.6 ± 0.9	37.1 ± 6.1	73.2 ± 3.1	8.3 ± 0.9	0.11 ± 0.01
3	0	100	0.75	21.0 ± 2.4	46.6 ±5.4	84.7 ± 4.7	24.0 ± 3.7	53.1 ± 5.9	86.4 ± 2.6	11.8 ± 1.6	0.02 ± 0.00
4	100	0	0.3	22.7 ± 2.1	52.1 ± 3.7	81.1 ± 3.7	26.6 ± 1.3	50.0 ± 4.8	85.5 ± 4.1	13.1 ± 2.2	0.04 ± 0.00
5	0	100	1.2	15.7 ± 1.6	44.2 ± 6.8	71.6 ± 4.2	21.0 ± 2.7	46.2 ± 3.6	79.0 ± 2.1	12.9 ± 1.4	0.02 ± 0.00
6	75	25	0.525	18.5 ± 2.6	45.9 ± 1.1	73.7 ± 6.1	25.6 ± 3.7	44.3 ± 2.9	81.9 ± 3.5	9.6 ± 0.8	0.08 ± 0.01
7	100	0	0.75	19.1 ± 3.7	39.5 ± 3.1	62.1 ± 3.7	25.5 ± 0.7	44.6 ± 3.4	76.8 ± 1.5	10.9 ± 1.8	0.10 ± 0.02
8	100	0	0.75	19.3 ± 2.6	42.2 ± 4.3	65.3 ± 5.8	25.1 ± 3.1	45.8 ± 5.9	75.4 ± 2.6	11.2 ± 2.1	0.10 ± 0.01
9	100	0	0.3	23.6 ± 4.1	50.8 ± 6.8	78.4 ± 7.7	26.1 ± 2.5	50.4 ± 5.1	85.9 ± 3.7	12.4 ± 1.5	0.04 ± 0.00
10	25	75	0.525	19.6 ± 3.6	47.3 ± 5.1	83.2 ± 6.8	24.6 ± 2.9	48.7 ± 4.6	84.5 ± 5.1	10.8 ± 0.9	0.02 ± 0.00
11	100	0	1.2	14.6 ± 2.2	39.2 ± 3.7	58.1 ± 1.9	21.5 ± 1.6	40.9 ± 3.3	67.2 ± 3.5	9.5 ± 0.6	0.14 ± 0.03
12	50	50	0.75	22.6 ± 3.8	44.4 ± 5.9	74.6 ± 5.3	25.3 ± 3.2	40.4 ± 4.9	81.8 ± 4.6	9.7 ± 1.4	0.07 ± 0.01
13	25	75	0.975	20.5 ± 2.1	42.3 ± 3.7	58.6 ± 3.6	23.2 ± 2.6	46.8 ± 5.7	79.0 ± 4.3	10.9 ± 0.7	0.01 ± 0.00
14	25	75	0.3	23.9 ± 2.8	58.9 ± 4.7	94.1 ± 4.1	26.8 ± 1.1	56.4 ± 6.2	89.8 ± 5.2	11.0 ± 0.8	0.03 ± 0.00
15	100	0	1.2	16.9 ± 3.1	39.5 ± 4.1	62.1 ± 2.9	21.5 ± 0.9	42.8 ± 1.6	67.4 ± 3.1	9.9 ± 1.3	0.14 ± 0.03
16	0	100	1.2	16.2 ± 2.6	45.0 ± 3.9	78.3 ± 3.3	22.4 ± 2.7	50.5 ± 2.5	80.3 ± 2.1	13.3 ± 1.5	0.02 ± 0.00
17	75	25	0.975	21.7 ± 2.1	38.3 ± 2.6	70.8 ± 5.6	24.4 ± 3.5	40.3 ± 5.9	73.2 ± 1.2	8.7 ± 0.7	0.17 ± 0.03
18	0	100	0.3	24.3 ± 4.1	61.4 ± 4.7	91.0 ± 6.9	26.5 ± 3.9	60.8 ± 4.1	89.6 ± 4.7	12.0 ± 1.1	0.01 ± 0.00
19	50	50	0.3	26.1 ± 2.2	55.1 ± 3.4	86.7 ± 5.8	27.0 ± 3.1	52.6 ± 4.8	89.2 ± 3.9	11.0 ± 0.7	0.04 ± 0.00

**Table 6:** Predicted and actual values of suggested solutions.

Factors	Responses	95% CI Low Predicted Value	Predicted Value	95% CI High Predicted Value	Observed Value	Error Percentage (%)
X_1_: 41.02%	Y_1_	23.1	24.2	25.2	24.9 ± 2.2	2.82
	Y_2_	55.8	56.8	57.9	57.7 ± 4.1	1.56
	Y_3_	84.5	89.5	94.6	91.4 ± 3.6	2.08
X_2_: 58.98%	Y_4_	26.4	26.9	27.4	26.4 ± 1.4	1.90
	Y_5_	49.1	51.8	54.6	54.3 ± 3.7	4.61
	Y_6_	87.7	88.4	89.1	88.6 ± 5.8	0.23
X_3_: 0.3	Y_7_	10.1	10.7	11.3	11.2 ± 1.2	4.47
	Y_8_	0.032	0.036	0.041	0.040 ± 0.004	10.0

**Table 7:** Parameters of Korsmeyer–Peppas model for GA and BMUL.

Drug	*n*	*k*	R^2^	Release Mechanism
GA	0.84	0.08	0.99	Non-Fickian
BMUL	0.89	0.07	0.98	Case II

## Supplementary Materials

The following are available online at https://www.mdpi.com/1999-4923/12/10/932/s1. Table S1: Analysis of variance for model of GA %dissolved at 2 h (Y_1_), Table S2: Analysis of variance for model of GA %dissolved at 6 h (Y_2_), Table S3: Analysis of variance for model of GA %dissolved at 12 h (Y_3_), Table S4: Analysis of variance for model of BMUL %dissolved at 2 h (Y_4_), Table S5: Analysis of variance for model of BMUL %dissolved at 6 h (Y_5_), Table S6: Analysis of variance for model of BMUL %dissolved at 12 h (Y_6_), Table S7: Analysis of variance for model of hardness (Y_7_), Table S8: Analysis of variance for model of friability (Y_8_).
