# Supplementary material for: Statistical Design of Sustained-Release Tablet Garcinia cambogia Extract and Bioconverted Mulberry Leaf Extract for Anti-Obesity
Source: Pharmaceutics. 2020 Sep 29;12(10):932. doi: 10.3390/pharmaceutics12100932 (PMC7600061; doi:10.3390/pharmaceutics12100932)
Supplement: Supplementary file 1 [file pharmaceutics-12-00932-s001.pdf]

pharmaceutics

Supplementary Material: Statistical Design of Sustained-Release Tablet *Garcinia cambogia* Extract and Bioconverted Mulberry Leaf Extract for Anti-Obesity

Hye-Jin Lee, Young-Guk Na, Mingu Han, Thi Mai Anh Pham, Hyeonmin Lee, Hong-Ki Lee, Chang-Seon Myung, Joo-Hui Han, Jong-Seong Kang, Kyung-Tae Kim, and Cheong-Weon Cho

Table S1. Analysis of variance for model of GA %dissolved at 2 h (Y_1).

Source	Sum of squares	df	F-value	p-value
Model	213.72	3	48.59	<0.0001
X_3	10.12	1	6.90	0.0190
X_3^2	1.12	1	0.8230	0.3787
X_3^3	38.40	1	26.19	0.0001
Residual	21.99	15		
Lack of fit	18.79	10	2.93	0.1234
Pure error	3.20	5		
Total	235.71	18		

Note: df (Degree of freedom), A large F-value implies a large impact on the modeling profile; A p-value of less than 0.05 means that it affects modeling profile.

Table S2. Analysis of variance for model of GA %dissolved at 6 h (Y_2).

Source	Sum of squares	df	*F*-value	*p*-value
Model	974.36	5	138.58	<0.0001
Linear mixture	208.62	1	148.35	<0.0001
X_1X_3	172.84	1	122.91	<0.0001
X_2X_3	338.80	1	240.93	<0.0001
$X_1X_3^2$	35.55	1	25.28	0.0002
$X_2X_3^2$	61.30	1	43.59	<0.0001
Residual	18.28	13		
Lack of fit	13.21	8	1.63	0.3064
Pure error	5.07	5		
Total	992.64	18		

Note: df (Degree of freedom), A large *F*-value implies a large impact on the modeling profile; A *p*-value of less than 0.05 means that it affects modeling profile.

Table S3. Analysis of variance for model of GA %dissolved at 12 h (Y_3).

**Source**	**Sum of squares**	**df**	***F*-value**	***p*-value**
Model	2627.82	7	30.55	<0.0001
Linear mixture	813.80	1	66.23	<0.0001
X_1X_2	58.43	1	4.75	0.0518
X_1X_3	388.83	1	31.64	0.0002
X_2X_3	383.71	1	31.23	0.0002
$X_1X_2X_3$	30.95	1	2.52	0.1408
$X_1X_2(X_1 - X_2)$	104.19	1	8.48	0.0141
$X_1X_2X_3(X_1 - X_2)$	156.78	1	12.76	0.0044
Residual	135.17	11		
Lack of fit	72.73	6	0.9706	0.5238
Pure error	62.44	5		
Total	2762.99	18		

Note: df (Degree of freedom), A large *F*-value implies a large impact on the modeling profile; A *p*-value of less than 0.05 means that it affects modeling profile.

Table S4. Analysis of variance for model of BMUL %dissolved at 2 h (Y_4).

**Source**	**Sum of squares**	**df**	***F*-value**	***p*-value**
Model	72.60	1	140.55	<0.0001
X_3	72.60	1	140.55	<0.0001
Residual	8.78	17		
Lack of fit	6.60	12	1.26	0.4257
Pure error	2.18	5		
Total	81.38	18		

Note: df (Degree of freedom), A large F-value implies a large impact on the modeling profile; A p-value of less than 0.05 means that it affects modeling profile.

Table S5. Analysis of variance for model of BMUL %dissolved at 6 h (Y_5).

Source	Sum of squares	df	*F*-value	*p*-value
Model	796.01	5	40.71	<0.0001
Linear mixture	255.30	1	65.28	<0.0001
X_1X_2	146.95	1	37.57	<0.0001
X_1X_3	69.70	1	17.82	0.0010
X_2X_3	175.49	1	44.87	<0.0001
$X_1X_2X_3$	6.20	1	1.58	0.2302
Residual	50.84	13		
Lack of fit	37.85	8	1.82	0.2638
Pure error	12.99	5		
Total	846.85	18		

Note: df (Degree of freedom), A large F-value implies a large impact on the modeling profile; A p-value of less than 0.05 means that it affects modeling profile.

Table S6. Analysis of variance for model of BMUL %dissolved at 12 h (Y_6).

Source	Sum of squares	df	*F*-value	*p*-value
Model	932.35	3	381.51	<0.0001
Linear mixture	264.03	1	324.12	<0.0001
X_1X_3	416.31	1	511.06	<0.0001
X_2X_3	148.83	1	182.70	<0.0001
Residual	12.22	15		
Lack of fit	10.00	10	2.26	0.1907
Pure error	2.21	5		
Total	944.56	18		

Note: df (Degree of freedom), A large F-value implies a large impact on the modeling profile; A p-value of less than 0.05 means that it affects modeling profile.

Table S7. Analysis of variance for model of hardness (Y_7).

**Source**	**Sum of squares**	**df**	***F*-value**	***p*-value**
Model	35.97	5	34.92	<0.0001
Linear mixture	5.38	1	36.09	0.0002
X_1X_2	17.30	1	83.98	<0.0001
X_1X_3	9.28	1	45.06	<0.0001
X_2X_3	0.9074	1	4.40	0.0560
$X_1X_2X_3$	0.7930	1	3.85	0.0715
Residual	2.68	13		
Lack of fit	2.15	8	2.53	0.1604
Pure error	0.5300	5		
Total	38.65	18		

Note: df (Degree of freedom), A large F-value implies a large impact on the modeling profile; A p-value of less than 0.05 means that it affects modeling profile.

Table S8. Analysis of variance for model of friability (Y_8).

Source	Sum of squares	df	*F*-value	*p*-value
Model	0.0470	11	686.59	<0.0001
Linear mixture	0.0242	1	3888.41	<0.0001
X_1X_2	0.0001	1	22.58	0.0021
X_1X_3	0.0101	1	1622.24	<0.0001
X_2X_3	0.0001	1	17.21	0.0043
$X_1X_2X_3$	0.0002	1	26.24	0.0014
$X_1X_3^2$	0.0001	1	22.07	0.0022
$X_2X_3^2$	0.0000	1	3.64	0.0982
$X_1X_2(X_1 - X_2)$	0.0015	1	241.59	<0.0001
$X_1X_2X_3^2$	0.0001	1	11.52	0.0115
$X_1X_2X_3(X_1 - X_2)$	0.0015	1	233.93	<0.0001
$X_1X_2X_3^2(X_1 - X_2)$	2.626E-07	1	0.0422	0.8431
Residual	0.0000	7		
Lack of fit	0.0000	2		
Pure error	0.0000	5		
Total	0.0471	18		

Note: df (Degree of freedom), A large F-value implies a large impact on the modeling profile; A p-value of less than 0.05 means that it affects modeling profile.

© 2020 by the author. Licensee MDPI, Basel, Switzerland. This article is an open access article distributed under the terms and conditions of the Creative Commons Attribution (CC BY) license (http://creativecommons.org/licenses/by/4.0/).
